# A Remotely Delivered, Personalized Music Therapy Pilot Intervention for Lonely Older Adults During the Covid-19 Pandemic

**DOI:** 10.1016/j.osep.2024.03.001

**Published:** 2024-04-16

**Authors:** Nichola R. Haddad, Twisha Bhardwaj, Benjamin S. Zide, Hema Kher, Jessica M. Lipschitz, Maria A. Hernandez, Suzanne B. Hanser, Nancy Donovan

**Affiliations:** Department of Psychiatry (NRH, JML), Brigham and Women’s Hospital, Harvard Medical School, Boston, MA; Division of Geriatric Psychiatry, Department of Psychiatry (TB, BSZ, MAH, ND), Brigham and Women’s Hospital, Harvard Medical School, Boston, MA; Department of Psychiatry (HK), Emory St. Joseph’s Hospital, Atlanta, GA; and the Berklee College of Music (SBH), Boston, MA. Send correspondence and reprint requests to: Nancy J. Donovan, M.D., 60 Fenwood Road 4120, Boston, MA 02115.

**Keywords:** music therapy, loneliness, Covid-19, music listening

## Abstract

**Introduction:**

This study investigated a remotely delivered, therapist-facilitated, personalized music listening intervention for community-dwelling older adults experiencing loneliness during the Covid-19 pandemic. We assessed its feasibility and individuals’ experiences of social connection and emotional well-being during the intervention.

**Methods:**

Ten cognitively unimpaired older adults who endorsed loneliness completed eight weekly sessions with a board-certified music therapist via Zoom. Participants were guided in developing two online personalized music playlists and were asked to listen to playlists for at least one hour daily. Feasibility metrics were attendance, accessibility, and compliance rates. Post-study interview responses were analyzed using a rapid qualitative methodology. Exploratory pre- and post-study measures of loneliness and other aspects of psychological well-being were obtained using validated questionnaires.

**Results:**

Ten participants (mean age 75.38 [65 to 85] years, 80% women) were enrolled from March to August 2021. Attendance and compliance rates were 100% and the accessibility rate was 90%. Most participants associated music with positive memories before the program and many reported that the intervention prompted them to reconnect with music or listen to music with greater intention. They cited increased connection from interacting with the music therapist and the music itself, as well as specific positive emotional impacts from integrating music into their daily lives. Median pre- to post-questionnaire measures of psychological function all changed in an improved direction.

**Discussion:**

Remotely delivered music therapy may be a promising intervention to promote regular music listening and socioemotional well-being in lonely older adults.

## INTRODUCTION

Enforced isolation during the Covid-19 pandemic has called attention to the pervasive problem of loneliness, particularly for older adults. Loneliness is subjective in nature and refers to the perceived discrepancy between one’s actual and desired level of social connection.^1^ Though conceptually distinct, loneliness is partially influenced by social isolation, a related construct which indicates the objective absence or limited extent of relationships or interactions with other people.^2^ Loneliness, in turn, is a risk factor for important health outcomes including cardiovascular and cerebrovascular disease, depressive and anxiety disorders, dementia and premature death.^3−5^ Prior to the Covid-19 pandemic, a nationally representative survey of older Americans found that 43% of older adults reported feelings of loneliness, including 13% of respondents who reported feeling lonely frequently.^6^

Levels of loneliness increased among many older adults during the pandemic and were associated with worsening anxiety and depression.^7−9^ For many, Covid-19 restrictions amplified the isolating implications of living alone, a risk factor for loneliness for both men and women.^10^ This risk was particularly pronounced among women living alone.^10^ While many older adults used digital platforms to maintain social contact and resilience to loneliness,^11^ virtual socialization appeared to be less effective as a substitute for in-person interaction for other older adults.^7^ Some research groups have looked beyond the use of digital platforms as a medium to promote social interaction, but, rather, to teach behavioral interventions that can be integrated into daily life to reduce loneliness and promote mental health.^12^

Music therapy is the clinical and evidence-based use of music interventions to accomplish individualized goals within a therapeutic relationship by a credentialed professional. Music therapy, including music listening interventions, has been shown to be effective for the treatment of mental health disorders,^13^ particularly depression and anxiety,^14^ and for the management of neuropsychiatric symptoms of dementia.^15^

Music listening is a natural and common pastime that is associated with improved affect and mood regulation, feelings of social connection, and with greater eudemonic well-being in older adults.^16−18^ Moreover, observational research supports that older adults listen to music to achieve these specific effects such as relaxation, reminiscing, and compensation for loneliness, qualitatively similar to studies of younger individuals.^18,19^ However, the intensity of listening decreases with age and older adults report the need for greater, more varied, and more personalized opportunities for listening and engagement with music.^19^

The known positive effects of music listening have not been broadly recognized in the medical literature as a primary or secondary preventative health strategy to promote socioemotional well-being for older adults, nor explicitly, as a strategy to cope with acute or severe life stressors. In response to the COVID-19 pandemic, we assembled a team of music therapy specialists along with clinicians and staff from a geriatric psychiatry clinical division to develop a very early-stage, therapist-facilitated, music listening intervention to advance this goal.

In this pilot study, we adapted a telephone-based music therapy intervention for clinical depression to a video-delivered format, for use as a behavioral intervention to support socioemotional well-being during the Covid-19 pandemic. This intervention aimed to foster regular, personalized music listening, facilitated by a qualified music therapist. We used attendance, accessibility, and compliance metrics to evaluate the feasibility of this video-based, eight-week personalized music therapy intervention for older adults endorsing loneliness. Additionally, we employed qualitative analysis of post-intervention interviews to examine the impact of the intervention on participant experiences of music-listening, social connection, and emotional well-being. To complement qualitative findings, exploratory pre-and post-study measures of loneliness and other aspects of psychological well-being were obtained using validated questionnaires.

## MATERIALS AND METHODS

### Participants

Community-dwelling older adults were recruited through an online research registry and clinician referrals between March and August 2021. Participants were age 65 years or older, English-speaking, endorsed feelings of loneliness based on a single-item loneliness measure from the Center for Epidemiological Studies-Depression (CES-D) scale, and were able to use videoconferencing.^20^ Individuals were excluded for hearing impairment, cognitive impairment based on a Telephone Interview for Cognitive Status (TICS) score < 31,^21^ or for moderate to severe depression based on a score of 10 or greater on the Geriatric Depression Scale (GDS), 15-item version.^22^

The Partners Human Research Committee approved this study, and all participants provided informed consent prior to enrollment.

### The Personalized Music Playlist Intervention

Participants completed eight weekly music therapy sessions conducted virtually using the Zoom video platform. This protocol was adapted from an evidence-based, telephone-delivered music therapy strategy that significantly reduced symptoms of depression in clinically depressed older adults.^23^ This personalized music playlist intervention was adapted to a videoconferencing-based format and was applied as a behavioral intervention to support socioemotional well-being during the Covid-19 pandemic rather than the treatment of clinical depression.

During the initial music therapy session, participants worked with a board-certified music therapist to curate “Relaxing” and “Energizing” playlists on YouTube Music. Participants listened to some of their music selections with the music therapist and discussed why the song was meaningful to them. They identified how the song made them feel, in order to categorize them into the two playlists. Participants were able to select music from any genre. Participants who were not able to finish putting together a playlist within a single one-hour session were offered the option of a second one-hour session prior to follow-up sessions. They were instructed to listen to their music playlists for at least one hour daily throughout the eight-week study, with flexibility to break up the music listening throughout the day. Participants were also encouraged to complete a daily journal, though this was not required. During each weekly 30-minute session, participants worked with the music therapist to edit and enhance playlists. Participants were offered the opportunity to learn music listening techniques, such as music-facilitated breathing, progressive muscle relaxation, and imagery, to incorporate into their daily music listening.

Music therapy sessions were tracked and prompted by the music therapist. For this study, the additional component of questionnaire administration, tracking, and prompting was carried out by research staff. Older adults received a single $100 stipend after, and only if, they completed all eight music therapy sessions and post-study assessments. This stipend was determined in accordance with Mass General Brigham Human Research Committee policy of suggested prorated compensation for research participants.

### Feasibility Criteria

Feasibility of the intervention was assessed according to attendance, accessibility (i.e., usability of the digital music-listening platform), and compliance rates. *Attendance rate* was calculated as the proportion of participants who attended the eight required sessions with the music therapist. *Accessibility rate* was defined as the proportion of participants who were able to utilize the YouTube music-listening platform to listen to their playlists. *Compliance rate* was defined as the proportion of participants who listened to their playlists for at least six out of seven days of the week for the duration of the intervention.

### Qualitative Questions

After completing the eight-week intervention, older adult participants responded to five open-ended program evaluation questions. Older adults responded via a telephone call with a study psychiatrist. Five domains were probed using the following questions: *(1) What role has music played in your life? (2) Has this changed after participating in this music therapy project? (3) How has the COVID-19 pandemic impacted your feelings of connection with others? (4) Has music therapy impacted your feelings of connection with others? If so, how? (5) Has music therapy affected your feelings of emotional well-being? If so, how?*

### Qualitative Analysis

Meaning was extracted from the open-ended program evaluation responses through a rapid qualitative analysis approach, an efficient and well-validated tool for qualitative program evaluation.^24−26^ Research staff assembled a templated summary of each set of survey responses. These summaries were organized by domains that corresponded to the interview questions. Finally, templated summaries were used to create a matrix in which participants were included as rows and domains as columns. Through a series of discussions, the research team reached consensus on included themes within each domain.

### Standardized Questionnaires

We measured clinical outcomes using standardized assessments of loneliness and other aspects of psychological well-being. This was undertaken to collect preliminary data on outcomes as a complement to the qualitative analyses and were exploratory in nature. Participants completed pre-, mid- and post-intervention assessments by answering online questionnaires at baseline and after the fourth and eighth music therapy session, using the RedCap Platform (RedCap, version 12.0.19). A RedCap link was emailed to participants who completed the questionnaires at home. Loneliness was assessed using the 8-item PROMIS Social Isolation Scale—Short Form 8a (score range 8−40; with higher score indicating greater loneliness).^27^ The Behavioral Activation for Depression Scale - Short Form (BADS), a nine-item scale, was used to assess behaviors impacting change according to the behavioral activation treatment model (score range 0–54; with higher score indicating greater activation).^28^ Positive emotions and psychological well-being were assessed with the Positive Affect and Well-Being—Short Form (PAWB), a nine-item scale (score range 9−45; with higher score indicating better affect and well-being).^29^ The 10-item Perceived Stress Scale (PSS) was administered to measure self-appraised levels of stress during the past month (score range 0−40; with greater score indicating greater perceived stress).^30^ Clinically relevant psychological symptoms were assessed with the 53-item Brief Symptom Inventory (BSI), which consists of nine symptom dimensions (score range 0−212; with higher score indicating greater symptoms).^31^ Last, the ability to experience pleasure was assessed with an adapted form of the 14-item Snaith-Hamilton Pleasure Scale (SHAPS) (score range 0−52; with higher score indicating greater pleasure).^32^

### Statistical Analyses

Descriptive statistics for the sample and for the clinical questionnaire data were derived using R software (R, version 4.0.4 R Foundation for Statistical Computing, Vienna, Austria). Pre-study to post-study change scores were calculated. As this was a pilot evaluation of feasibility and qualitative aspects of participant experience, questionnaire measures were not evaluated with inferential statistics. For descriptive purposes, we considered that numerical pre-post improvement in the mean or median questionnaires scores for the sample may point towards a possible intervention effect.

## RESULTS

### Enrollment and Sample Characteristics

Of the 31 older adults who responded to the initial recruitment advertisement, 17 were reached for follow-up and underwent telephone screening. Seven individuals were not enrolled because of inclusion/exclusion criteria (medical exclusions [n = 1], did not endorse loneliness [n = 2]) or a decision against enrollment (n = 4). Meeting the recruitment target, the final sample was comprised of ten older adults with a mean age (SD) of 75.4 (6.7) years. The eight female participants were all unmarried (never married [n = 2], divorced [n = 3], or widowed [n = 3]) and lived alone. Two participants were men who lived with their spouses. At screening, the mean GDS score (SD) of the sample was 4.0 (3.5) and mean TICS score (SD) was 40.5 (2.92), indicating an absence of severe depressive symptoms and an absence of cognitive impairment, respectively.

### Attendance, Accessibility, and Compliance

All ten participants attended their eight required sessions with the music therapist, with two participants opting to receive an extra introductory session due to slower progress through the program. This reflects an *attendance rate* of 100% for the intervention. Further, all ten participants reported independently listening to their playlists at least six out of seven days of the week for all eight weeks, corresponding to a 100% *compliance rate* with the music listening protocol. Finally, nine out of ten participants were able to utilize the YouTube platform to create and listen to their curated playlists throughout the week, which we defined as a 90% *accessibility rate* for the intervention. Accommodation was made for one participant who listened to a preferred radio station instead of the online playlist, due to difficulties using YouTube Music independently. Five participants chose to learn optional music listening techniques during the music therapy sessions and applied these techniques to their daily listening.

### Qualitative Analysis

Interview questions covered five domains: the role of music in participants’ lives before the intervention, the role of music in participant’s lives after the intervention, the impact of Covid-19 on social connection, the impact of the intervention on social connection, and the impact of the intervention on emotional well-being. For each domain, themes are described below and summarized with illustrative quotations in Table 1.

#### Domain 1: Role of music in life before intervention

Most older adults reported that music was a part of *positive experiences and events* from earlier in their lives. Several participants reflected on the integral role of music in their formative years, explaining that their childhood homes and communities were often filled with music or that they learned to play musical instruments while growing up. For another participant, music at Girl Scout camp had served as an escape from her difficult home life. Additionally, many participants in this category fondly remembered major musical performances that they had attended at different points in their lives, including Broadway shows and concerts by bands like the Beatles.

#### Domain 2: Impact of music intervention

Many older adults reported that participating in the intervention prompted them to *reconnect with music* or to *listen to music with greater intention*. Participants discussed rediscovering artists or genres that they had enjoyed in the past and the novelty of exploring unfamiliar artists. Participants also described a heightened sense of mindfulness, viewing the intervention as an “opportunity to take advantage of focusing” on the ways that music listening affected them. Others explained that they purposefully used their distinct playlists to feel calmer or more energized for a given task, or even to regulate somatic effects, such as blood pressure, heart rate, and sleep.

#### Domain 3: Impact of COVID-19 on social connection

Participants described how the Covid-19 pandemic had a *varied impact on social connection*, citing negative, neutral, and positive experiences. For many participants, the social isolation enforced during the pandemic made them feel as if “there’s nothing” and contributed to strong feelings of loneliness, putting “a real strain” on the social connections and engagements that previously characterized their daily routine. Participants with lower baseline social contact did not notice any change in feelings of connection to others, feeling that even amid the pandemic, “life is as usual for me.” Some older adults reported a strengthened sense of social connection during the pandemic. These participants harnessed digital platforms such as WhatsApp, Zoom, and FaceTime not only to connect with family and friends, but also to engage in virtual classes and volunteering opportunities.

#### Domain 4: Impact of intervention on social connection

Most older adults cited *increased connection* during the intervention, from interacting with the music therapist and with the music itself. Many participants in this category described the “joy” they experienced from working with the therapist during their sessions, explaining how this form of stimulating, one-on-one engagement during the pandemic “helped some pretty empty days go by.” Others explained that the music itself was a form of “company” to them during solitary activities and even described a deep sense of companionship with the artist, feeling “hugged” while “spending the time with him.”

#### Domain 5: Impact of intervention on emotional well-being

Finally, participants noted a range of *positive emotional impacts* from integrating music into their daily lives. Some described the capacity of music to promote positive mood by “lift(ing) me up” or “get(ting) me energized.” Others noted how music often alleviated negative mood by “cheer(ing) me up. . . out of a depression” or helping them feel less “riled up in the morning and evening,” when they would have other-wise been watching the news.

### Clinical Outcomes

**FIGURE 1.**
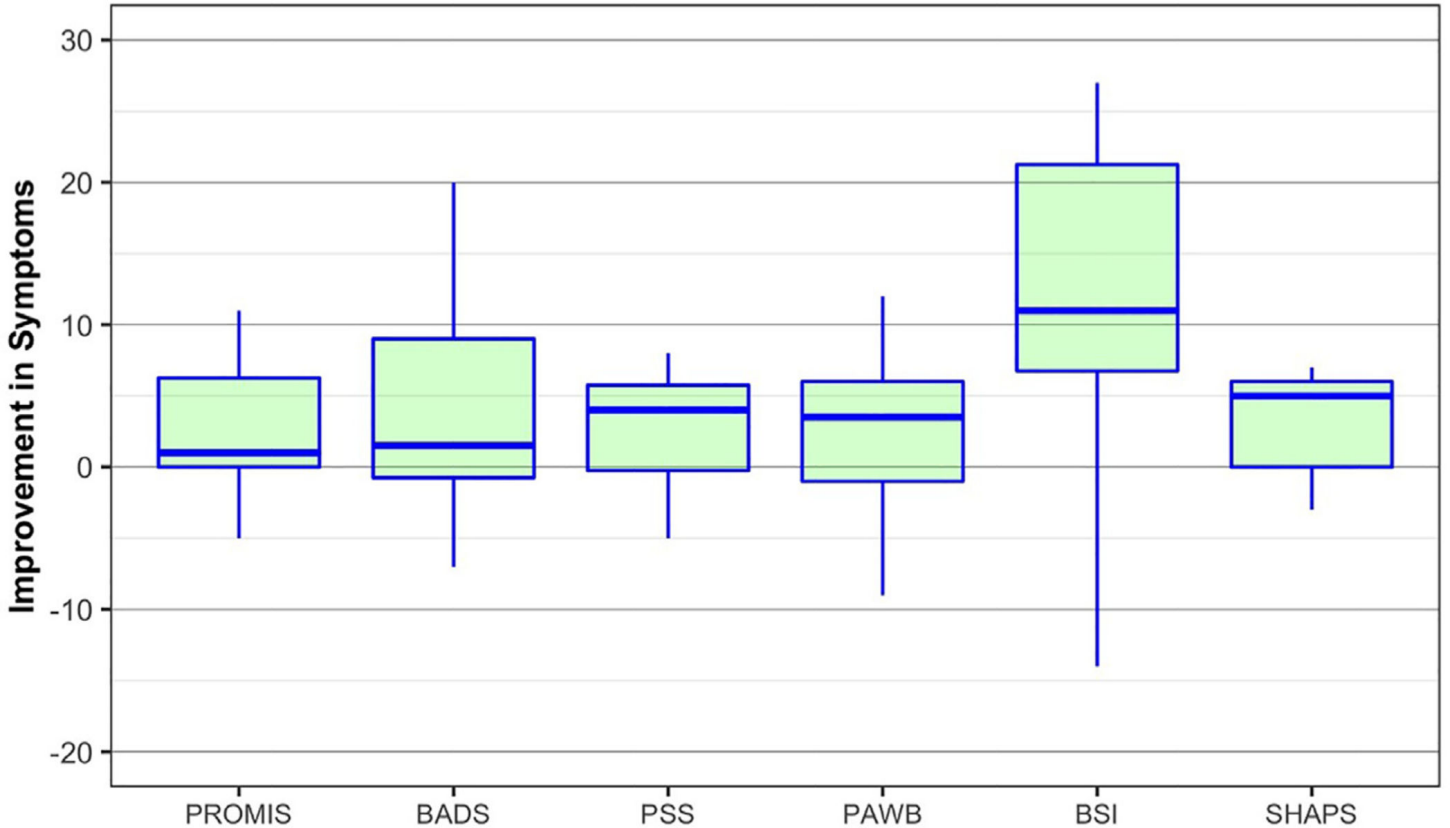
Pre- to post-study changes in clinical outcomes for the sample. Each box plot indicates the median and interquartile range for the ten older adult participants who completed both baseline and endpoint questionnaires. Change score values were calculated for each measure by subtracting a participant’s endpoint score from their baseline score, where a positive change score indicates an improvement in symptoms. Abbreviations: PROMIS: patient-reported outcomes measurement information system (social isolation short form); BADS: behavioral activation for depression; PSS: perceived stress scale; PAWB: positive affect and well-being; BSI: Brief Symptom Inventory; SHAPS: Snaith-Hamilton Pleasure Scale.

Data from the clinical questionnaires are presented in Figure 1. For the sample, median pre- to post-study change scores for loneliness, behavioral activation, positive affect and well-being, perceived stress, and pleasure all lie in a positive (improved) range (Fig. 1). These exploratory data are presented in greater detail in the supplement online.

## DISCUSSION

This study evaluated the feasibility and participant experiences of social connection and emotional well-being during a remotely delivered, therapist-facilitated personalized music listening intervention during the Covid-19 pandemic. The overall feasibility of the intervention was supported by high rates of attendance, accessibility, and compliance. While nine out of ten participants learned to utilize YouTube Music to curate and listen to their playlists, one participant was not able to access the online platform independently and relied on a favorite radio station for music listening. All participants engaged in the eight video-based sessions with the music therapist and reported independently listening to their personalized music for at least one hour, nearly every day. Thus, this remotely delivered music therapist intervention was effectively leveraged, and in one case adapted, to teach and encourage older adults to integrate music into their daily routines and lives.

Our qualitative data indicated that before the intervention, most older adults had positive memories of music during childhood, young adulthood, and significant musical events including concerts and theater productions. Some explained that music played less of a role later in their lives due to health problems or that music simply “fell out of my life.” The intervention enabled many participants to reestablish or reshape their relationship with music. It also represented an opportunity to enjoy music with greater intention and a heightened awareness of its physical effects and mindfulness benefits.

While social restrictions during the Covid-19 pandemic affected populations overall, their impacts on individuals were heterogeneous.^8,10,33^ Older adults in this study described personal histories and living circumstances that shaped varied pandemic experiences of social connection. Responses ranged from an urgent desire for company to an experience in which the pandemic “slowed things down” and allowed for greater social participation. Some older adults discussed new ways of maintaining social connection during this time, from holding Shabbat with friends via Zoom to joining virtual tai chi classes and messaging on WhatsApp.

In the midst of altered social dynamics during the pandemic, the music therapy intervention exerted either a neutral or positive influence on feelings of connection. Many participants described the positive experience of interacting with the music therapist during weekly sessions or a perception of music itself as a companion. Others noted that the music intervention made them more comfortable reaching out to others and served as a topic of conversation with friends. Notably, most participants described how integrating music listening into their quotidian lives enhanced their mood. These emotional reactions manifested as feelings of calmness and relaxation, or joyous and energizing effects from the playlists. While this pilot study was not powered for inferential statistics, quantitative measures of psychological function showed more favorable ratings after the intervention, consistent with these qualitative responses.

These findings add to observational research which demonstrates that people naturally listen to music to enhance their mood. In a cross-sectional study of 550 community-dwelling Swedish older adults prior to the Covid-19 pandemic, Laukka found that the most common responses to music listening were emotions and feelings of happiness, enjoyment, nostalgia, and relaxation.^17^ Correspondingly, participants who listened to music had better scores on measures of positive and negative affect and for personal growth, a dimension of eudemonic well-being encompassing self-efficacy and self-actualization. In a comprehensive study of 129 proposed functions of music listening derived from the extant literature, Schafer and colleagues surveyed responses from 834 German study participants ranging from age 8 to 85 years.^18^ While three distinct functions for music listening (consistent across age groups) were suggested by principal component analysis, the strongest component corresponded to music as a means to regulate mood and arousal by creating a positive mood or relaxing or energizing states. Our study suggests that music might be more widely and strategically deployed to support emotional well-being under conditions of social isolation or life stress. Moreover, the guidance of a trained music therapist may be a critical component to promote and realize the full benefits of music listening.

There are limitations to this study. A stipend was provided as compensation after, and only if, participants completed all eight music therapy sessions and post-study assessments, which introduced a potential favorable bias for one of our feasibility metrics (attendance rate). Second, as the quantitative measures were intended as descriptive data to complement qualitative methods, the small sample size was insufficient for statistical analyses of quantitative outcomes. Further, given the lack of randomization and a control condition, favorable trends in these outcomes could be attributable to changes in social distancing behaviors and/or improvement in health statistics with increased rates of vaccination or other non-intervention factors. Variability in the use of specific music listening techniques by participants was not considered in the interpretation of participant responses to the main intervention. Moreover, responses to the intervention may have been driven by the participant-therapist relationship and interactions independent of the music listening component. It is noteworthy that nearly all study participants were unmarried women who were living alone. Though this represents a high-risk group for loneliness,^10^ it limits the generalizability of our findings to older adults more broadly. Importantly, older women are a demographic group at particularly high risk for late-life depression and dementia, especially in the setting of social isolation, such as living alone or spousal loss, and loneliness.^34^ Thus, music listening may have special value as a lifestyle intervention for older women who live alone. Finally, this study was a pilot study so increasing the sample size was beyond scope. Thus, while we did see substantial repetition of key themes across participants, sample size was determined by practical limitations rather than a determination that data saturation was reached.

In conclusion, this remotely delivered personalized music listening intervention was met with high attendance, accessibility, and compliance rates. We found qualitative evidence for enhanced feelings of connection and emotional benefits from both the independent playlist-listening and therapist-interactive components of the program. Remotely delivered music therapy merits further investigation as a novel behavioral approach to support regular music listening and socioemotional well-being in older adults experiencing social isolation or loneliness in contexts beyond the pandemic.

## Supplementary Material

Supplement

## Figures and Tables

**TABLE 1.** Themes and Representative Quotations From Qualitative Analysis Responses by Intervention Participants

Theme	Example Quotations
**Domain 1: Role of music in life before intervention**	
*Music evoked positive memories and thoughts*	“I’ve always loved music, even when I was a little kid... There was a lot of music in the house. And when I was 16, we went to Woodstock... When I was 12 my best friend’s parents took us to the Ed Sullivan Show to see the Beatles, and we were in the third row. The minute I get in the car I turn on the radio. There’s always music. I can’t even drive if there isn’t music. A lot of these songs brought back all these great memories of when I lived in Miami Beach and Fort Lauderdale.” (Participant 5) “I had a troubled family life and got to go away to Girl Scout camp. Particularly the Girl Scout songs were very important to me. They were an important escape from difficult times...” (Participant 3)
**Domain 2: Impact of music intervention**	
*Reconnecting with music*	“The project really helped reconnect me. I even have one of my playlists in the background right now.” (Participant 10) “In this way, I connected again with a lot of music from my country from Romania. Even classical music which I ignore. I have the discs, but I ignore it. But this brought me back to that...” (Participant 1)
*Listening to music with intention*	“This really helped me [become] even more aware of how it affects me. It gave me the opportunity to take advantage of focusing on music... Reminds me of all the mindfulness classes I take at Dana [Farber] on Zoom. One of them was focusing on eating a raisin and really focusing in. So, it’s kind of like that but for music.” (Participant 5) “Well, what changed was making the separate lists. Before I wasnť thinking about that, but the advice to make different lists, calm, energizing, and now I have new lists of zen and classical music. I liked that very much. Looking for new songs I think is very beneficial. And that I think is the bigger benefit from this project. It’s more organized.” (Participant 1)
**Domain 3: Impact of COVID-19 on social connection**	
*Varied impact on connection*	“Oh lord I feel so isolated. I feel very lonely. I live on a second-floor apartment on a busy street. And I threaten to stick my head out of the window to call out to people to talk to me.” (Participant 3). “[COVID-19] hasn’t really affected my feelings of connection to others. I am what I am. I’m a creature of habit. Just the nature of what I’ve been contending with. I haven’t really mixed in. It hasn’t really had an effect. Life is as usual for me.” (Participant 2) “Actually, in some ways, [COVID-19 has] made it stronger with some people because I Zoom or Skype or WhatsApp with them. I’ve connected with some friends more because of COVID... I Zoom with some of my cousins in Israel that I would have never talked to if it wasn’t for COVID.” (Participant 4)
**Domain 4: Impact of intervention on social connection**	
*Connection with therapist*	“[Therapist] is a joy. Seeing her face light up when we were picking music and there were songs she hadn’t heard before that she enjoyed and seeing her face light up and enthusiasm. It was a really good connection and it cheered me up.” (Participant 3) “One of the nicest things about this project was getting to know [Therapist]. She is fantastic and exceptional. I really enjoyed working with her.” (Participant 4)
*Connection with music*	“I couldn’t get enough of Pavarotti. He was my constant companion. It was like having a companion play for me or sing for me... It enhanced my feeling of connection to music. I felt hugged by Pavarotti... This was something really special spending the time with him, or him spending the time with me.” (Participant 7) “I’m 81 years old. But I have a lot of music in my head. It keeps me company and I enjoy it while I walk. Songs from different parts of my life.” (Participant 9)
**Domain 5: Impact of intervention on emotional well-being**	
*Positive emotional impact from music intervention*	“I felt happier, and it could be the music because it was happy music. Overall, it had a positive impact on my emotional well-being. It became an interest. It was something fun to think about and discover more about.” (Participant 8) “Actually, listening to the music has been fun. And sometimes it cheers me up. The relaxing one will make me more relaxed and get me out of a depression. The exciting ones get me energized. The music therapy cheered me up.” (Participant 3)

## Data Availability

A portion of this work was presented at the American Association for Geriatric Psychiatric 2022 Annual Meeting, March 18–21, 2022, Orlando, FL, poster session: Kher H, Haddad N, Zide B, Hernandez M, Hanser S, Donovan N. “A Personalized, Telehealth Music Therapy Intervention for Lonely Older Adults: A Feasibility Study”. This work was also presented at the American Psychiatric Association’s Annual Meeting May 21–25, 2022, New Orleans, LA, poster session: Haddad N, Kher H, Zide B, Hernandez M, Donovan N. “A Personalized, Telehealth Music Therapy Intervention for Lonely Older Adults: A Feasibility Study”.

## Highlights

- What is the primary question addressed by this study?
  What is the feasibility and qualitative experience of a personalized music listening therapy intervention to promote socioemotional well-being for community-dwelling older adults during the Covid-19 pandemic?
- What is the main finding of this study?
  In this eight-week study, feasibility was supported by high attendance, compliance, and accessibility rates. Most participants reported that the intervention prompted them to reconnect with music or listen to music with greater intention, cited increased connection from interacting with the music therapist and the music itself, and experienced positive emotional impacts from integrating music into their daily lives.
- What is the meaning of the finding?
  Remotely delivered music therapy may be a promising intervention to promote regular music listening and socioemotional well-being in lonely older adults.
