# Supplementary material for: A Remotely Delivered, Personalized Music Therapy Pilot Intervention for Lonely Older Adults During the Covid-19 Pandemic
Source: Am J Geriatr Psychiatry. Author manuscript; Available in PMC 2025 Apr 16. (PMC11236479; doi:10.1016/j.osep.2024.03.001)
Supplement: Supplement [file NIHMS2002041-supplement-Supplement.docx]

**Supplementary Digital Content**

A remotely delivered, personalized music therapy pilot intervention for lonely older adults during the Covid-19 pandemic

Nichola R. Haddad, M.D., Twisha Bhardwaj, B.A., Benjamin S. Zide, M.St., Hema Kher, M.D., Jessica M. Lipschitz, Ph.D., Maria A. Hernandez, M.D., MT-BC, Suzanne B. Hanser, Ed.D., MT-BC, Nancy J. Donovan, M.D.

TEXT. Methods, Results and Discussion of clinical measures collected at baseline, midpoint, and endpoint of the study.

TABLE S1: Descriptive characteristics of pre-, mid-, and post-study scores.

FIGURE S1: Longitudinal changes in clinical outcomes for the sample.

This supplementary material has been provided by the authors to give readers additional information about their work.

**TEXT**

***Methods***

***Clinical Measures***

Quantitative scales were selected and administered to help characterize the social-emotional and psychological functioning of the sample during the study to complement the main qualitative analyses. We chose outcomes that would be expected to improve in domains relevant to music listening, based on prior observational research, in a non-clinical sample. These expected domains of improvement included loneliness, behavioral activation, positive affect and wellbeing, perceived stress, an inventory of psychological symptoms and level of pleasure.

We considered that numerical pre-post improvement in the mean or median score for the sample for each questionnaire could reflect a possible intervention effect. We further considered that a change of 0.5 standard deviation would also support a possible intervention effect. This change was estimated for each questionnaire by converting participant raw scores to z-scores, calculating the change in pre- post- z-scores for each participant and then calculating the mean value for the sample.

***Results***

Pre-, mid-, and post-study mean scores for study questionnaires are presented in Table S1. Pre- and post-study data were acquired for all 10 participants, while mid-study data were acquired for 9 participants. Mean z-score changes for each questionnaire are shown in Table S2. We provide spaghetti plots for each participant, acknowledging that the study was small and non-randomized, and that individual-level differences and COVID-related factors likely contributed to questionnaire responses.

There were numerical improvements in mean scores for all six outcome measures from baseline to study midpoint, as well as from baseline to study endpoint (Table S1; Figure S1).

***Discussion***

These preliminary data point to possible improvements in socioemotional and psychological function for all measures based on approximately one-half standard deviation changes in scores on validated questionnaires. We provide spaghetti plots for each participant, noting that the study was small and non-randomized, and that individual-level differences and other influences, including COVID-19 factors, would be expected to contribute to variability in questionnaire responses.

***TABLE S1. Pre-, mid-, and post-study scores for the standardized questionnaires.***

| **Measure**  (possible range) | **Pre-study mean (SD)**  (n=10) | **Mid-study mean (SD)**  (n=9) | **Post-study mean (SD)**  (n=10) |
| --- | --- | --- | --- |
| **PROMIS**  (8-40) | 20.4 (5.34) | 16.89 (4.34) | 18.0 (3.53) |
| **BADS**  (0-54) | 34.30 (10.72) | 41.44 (5.75) | 38.60 (9.80) |
| **PAWB**  (9-45) | 33.40 (6.00) | 36.44 (5.73) | 36.00 (5.10) |
| **PSS**  (0-40) | 15.30 (7.20) | 9.89 (6.74) | 12.5 (4.86) |
| **BSI**  (0-212) | 34.60 (21.60) | 23.80 (16.60) | 23.00 (15.10) |
| **SHAPS**  (0-52) | 46.40 (7.69) | 48.78 (6.02) | 49.7 (5.54) |

**Abbreviations:** PROMIS, Patient-Reported Outcomes Measurement Information System (Social Isolation Short Form**)**, higher score indicates greater loneliness**;** BADS, Behavioral Activation for Depression, higher score indicates greater activation; PAWB; Positive Affect and Well-being, higher score indicates better affect and well-being; PSS, Perceived Stress Scale, higher score indicates greater stress; BSI, Brief Symptom Inventory, higher score indicates more symptoms; mean item score for 212 items rather than total score is shown; SHAPS, Snaith-Hamilton Pleasure Scale, higher score indicates greater pleasure. The mean and standard deviation (SD) are shown.

***TABLE S2. Pre- post- changes in z-scores for standardized questionnaires.***

| **Measure**  (possible range) | **z-score change in improved direction (n=10)** |
| --- | --- |
| **PROMIS**  (8-40) | 0.53 |
| **BADS**  (0-54) | 0.46 |
| **PAWB**  (9-45) | 0.47 |
| **PSS**  (0-40) | 0.43 |
| **BSI**  (0-212) | 0.64 |
| **SHAPS**  (0-52) | 0.51 |

**Abbreviations:** PROMIS, Patient-Reported Outcomes Measurement Information System (Social Isolation Short Form**)**; BADS, Behavioral Activation for Depression; = Positive Affect and Well-being; PSS, Perceived Stress Scale; BSI, Brief Symptom Inventory; SHAPS, Snaith-Hamilton Pleasure Scale. The positive direction of z-scores indicates improvement in socioemotional or psychological function.

***FIGURE S1: Longitudinal changes in clinical outcomes for the sample.***


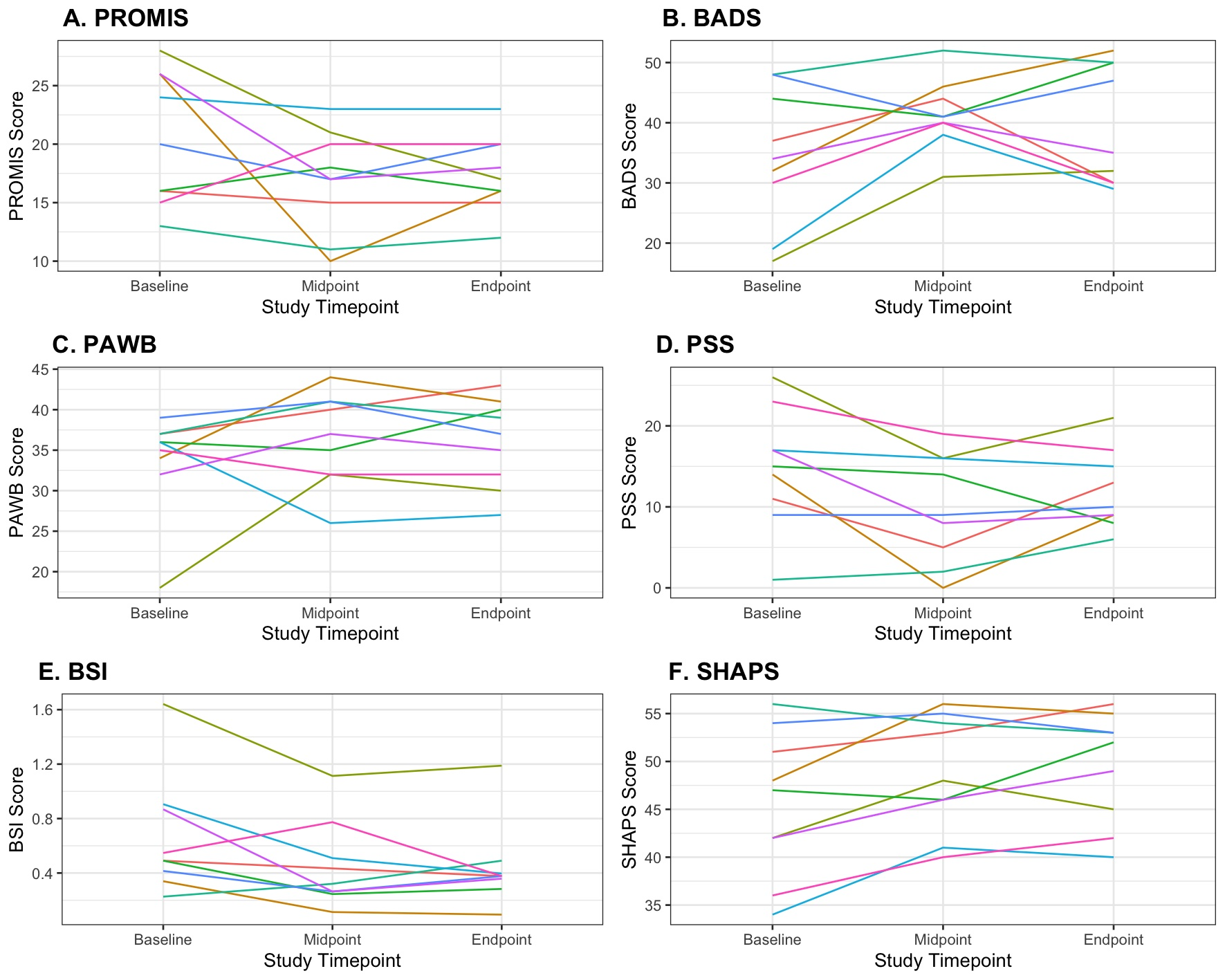


**LEGEND**

Each color represents scores for the same participant across all measures. Data are shown for 9 participants who completed baseline, midpoint, and endpoint study questionnaires. **A)** PROMIS, Patient-Reported Outcomes Measurement Information System (Social Isolation Short Form**)**, higher score indicates greater loneliness**; B)** BADS, Behavioral Activation for Depression, higher score indicates greater activation; **C)** PAWB; Positive Affect and Well-being, higher score indicates better affect and well-being; **D)** PSS, Perceived Stress Scale, higher score indicates greater stress; **E)** BSI, Brief Symptom Inventory, higher score indicates more symptoms; mean item score for 212 items rather than total score is shown; **F)** SHAPS, Snaith-Hamilton Pleasure Scale, higher score indicates greater pleasure.
